# Sociodemographic profile and access to health services among people attending the Transgender Outpatient Clinic: a cross-sectional study, Brazilian Federal District, 2018-2022

**DOI:** 10.1590/S2237-96222026v35e20251037.en

**Published:** 2026-08-03

**Authors:** Francisca Lidiane Sampaio Freitas, Ximena Pamela Díaz Bermúdez, Edgar Merchán-Hamann

**Affiliations:** 1 Universidade de Brasília Faculdade de Ciências da Saúde Brasília DF Brazil Universidade de Brasília, Faculdade de Ciências da Saúde, Brasília, DF, Brazil

**Keywords:** Transgender Persons, Transvestites, Access to Health Services, Primary Health Care, Cross-Sectional Studies, Personas Transgénero, Travestis, Acceso a los Servicios de Salud, Atención Primaria de Salud, Estudios Transversales

## Abstract

**Objectives:**

To characterize the sociodemographic profile, gender transition trajectory, and access to health services of trans persons and travestis, as well as to identify factors associated with not having been accessed by the Family Health Strategy (FHS).

**Methods:**

This was a cross-sectional study by means of analysis of medical records of trans persons and travestis (≥18 years) attending the Federal District Transgender Outpatient Clinic, 2018-2022. Descriptive analysis stratified by gender was performed. Associated factors were analyzed using bivariate analysis (prevalence ratio [PR] with 95% confidence interval [95%CI]) and multivariate analysis using binary logistic regression (adjusted odds ratio [OR] with 95%CI).

**Results:**

Out of 261 records (57.0% trans men, 37.6% trans women, and 5.4% travestis), the majority were young people (66.0%, 18-29 years old), Black (61.3%), with paid employment (46.7%) and complete high school or lower level education (54.3%); 25.0% had legally changed their name, 73.2% reported discrimination, and 49.0% of trans women and travestis began their transition without support. Mental disorders (46.4%) were the most frequently reported; and 17.2% had health insurance. Higher use of public urgent care and emergency services (56.7%) was observed compared to primary care (21.1%). Seeking care at a primary health care center when becoming ill decreased the odds of a negative outcome by 86.0% (OR 0.14; 95%CI 0.05; 0.37).

**Conclusion:**

Sociodemographic inequities, inequalities in gender transition trajectories, as well as in access to healthcare were evident, with underutilization of primary care. Being linked to a primary health care center proved to be a protective factor, emphasizing the FHS as essential for comprehensive care.

## Resumen

**Objetivos:**

Caracterizar el perfil sociodemográfico, la trayectoria de transición de género y el acceso a los servicios de salud de personas transgénero y travestis, así como identificar los factores asociados a la falta de acceso a la Estrategia de Salud Familiar (ESF).

**Métodos:**

Estudio transversal mediante el análisis de expedientes médicos de personas transgénero y travestis (≥18 años) atendidas en la Clínica de Atención Ambulatoria para Personas Transgénero del Distrito Federal, entre 2018 y 2022. Se realizó un análisis descriptivo estratificado por género. Los factores asociados se analizaron mediante análisis bivariado (razón de prevalencia [RP] con intervalo de confianza del 95 % [IC95%]) y análisis multivariado mediante regresión logística binaria (odds ratio [OR] ajustado con IC95%).

**Resultados:**

Entre 261 registros (57,0% hombres trans, 37,6% mujeres trans y 5,4% travestis), la mayoría eran jóvenes (66,0%, de 18 a 29 años), negros (61,3%), con empleo remunerado (46,7%) y con estudios secundarios o inferiores (54,3%); el 25,0% había cambiado legalmente su nombre, el 73,2% reportó discriminación y el 49,0% de las mujeres trans y travestis iniciaron su transición sin apoyo. Las enfermedades psiquiátricas (46,4%) fueron las más frecuentes; y el 17,2% tenía seguro médico. Se observó mayor utilización de los servicios públicos de urgencia y emergencia (56,7 %) en comparación con la atención primaria (21,1%). Acudir a un centro de atención primaria de salud al enfermar redujo la probabilidad de un desenlace negativo en 86,0% (OR 0,14; IC95%: 0,05; 0,37).

**Conclusión:**

Se evidenciaron inequidades sociodemográficas, desigualdades en las trayectorias de transición de género y en el acceso a la atención sanitaria, con una baja utilización de la atención primaria. La vinculación con el centro de atención primaria de salud demostró ser un factor protector, reforzando la ESF como esencial para una atención integral.

**Ethical aspects**

This research respected ethical principles, having obtained the following approval data:

**Research ethics committee** **Opinion number**, **Certificate of submission for ethical appraisal **, **Approval date**

Universidade de Brasília 6,333,242, 69644223.4.0000.0030, 29/9/2023

Secretaria de Saúde do Distrito Federal 6,650,598, 69644223.4.3002.5553, 15/2/2024

**Informed consent record ** Exempted.

## Introduction

The Brazilian Unified Health System (*Sistema Único de* Saúde, SUS) has achieved significant progress in providing care to the Brazilian population. However, political, programmatic, social and economic challenges persist that prevent its full implementation [1]. Access to health is not limited to one-off appointments, but constitutes a continuous process of care, conditioned by the effectiveness of care actions [2]. Comprehensive health care requires that services, at all levels of care, organize processes and practices to adequately meet demands [3].

The precepts of the National Primary Care Policy [4] define Primary Health Care (PHC) as the preferred entry point to the SUS, which must be free from any form of exclusion. The Family Health Strategy (FHS) is a PHC care model, focusing on illness prevention and health promotion and recovery. It is the responsibility of the Brazilian states and Federal District to strengthen it as a priority action, based on action in defined territories. A person having been accessed by the FHS indicates monitoring of a health situation or a home visit made by the FHS team, for example. This can reduce the demand for emergency services, prevent unnecessary hospitalizations and ensure referrals to specialized care.

In practice, ensuring access for transgender (trans) people to primary health care and specialized care services is a challenge. This population faces a reality marked by multiple barriers and systematic rights violations, especially when their health needs require comprehensive care that transcends the gender affirmation process [5].

In the Federal District, 1% of adults self-identified as trans in 2021 [6]. The reality of this population is marked by a contradiction: despite the existence of the Transgender Outpatient Clinic, which provides specialized care with a clinical and psychosocial approach, structural problems still persist, such as fragmentation of the care network, overloading of the service and limited PHC capacity to attend to trans people [7].

This study has proven itself to be relevant by filling gaps in research on care pathways (especially primary health care), as well as contributing to the development of more equitable health policies for trans people and transvestites (travestis) at all levels of care. Two scientific articles [8,9] produced in the context of the Transgender Outpatient Clinic were identified, but they address themes distinct from this research.

The objectives of this study were to: characterize the sociodemographic profile, gender transition trajectory, and health service access conditions of travestis, trans women, and trans men; and to identify factors associated with not having been accessed by the FHS.

## Methods

### Design 

This is a cross-sectional study conducted using data from the research project entitled “Project Athena: building evidence for action and decision-making on comprehensive health, infectious diseases and co-infections.” The project is a partnership between the University of Brasília and the Federal District Health Department Specialized Center for Infectious Diseases.

### Setting

This study was conducted at the Transgender Outpatient Clinic, part of Specialized Center for Infectious Diseases, created in 2017 [7]. However, the Outpatient Clinic was only accredited by the Ministry of Health to provide specialized care in August 2024 [10].

The main ways of accessing the service are: spontaneous demand; referral by the Specialized Social Assistance Reference Center (*Centro de Referência Especializado de Assistência Social*, CREAS) for Diversity; and referral by other social and health services. Reception carried out by the multidisciplinary team at the outpatient clinic upon patient arrival follows three stages: admission group; individual interview, using the reception form; and scheduling appointments according to needs [7].

### Participants

The convenience sample consisted of consecutive medical records of trans people and travestis, aged 18 or older, who began attending the outpatient clinic between 2018 and 2022. The year 2020 was excluded due to the disruptive impact of the COVID-19 pandemic and interruption of services at the clinic. This aimed to ensure that the analysis reflects the regular dynamics of access to services, without the distortion caused by this exceptional event.

### Variables

In order to ensure statistical robustness and maintain an adequate sample size for analyses of association, variables were recategorized using two main criteria: grouping categories with insufficient frequency and preserving statistical power in the analyses. This approach resulted in a distribution organized into three distinct blocks, as follows.

- Sociodemographic characteristics: age group at admission to the clinic (18-29, 30-39, 40-49, 50+ years), sexual orientation (heterosexual, homosexual, bisexual, other), race/skin color (mixed race, Black, White, Asian, Indigenous, unable to answer), marital status (single, married, stable union, divorced, widowed, preferred not to answer, unavailable/unknown), schooling (incomplete elementary education, complete elementary education, incomplete high school education, complete high school education, incomplete higher education, complete higher education, postgraduate education, unavailable/unknown), occupation (paid activity, sex work, unemployed, student, other, unavailable/unknown), welfare benefits (yes, no, unavailable/unknown) and housing situation (shelter/reception institution, street dweller, rented or free accommodation, own property, unavailable/unknown)
- Gender transition trajectory: prior knowledge of the Transgender Clinic (through friends or acquaintances, referral by a health service, social networks, Social Assistance Reference Center [*Centro de Referência de Assistência Social*, CRAS] and/or CREAS, other, unavailable/unknown), reason for seeking care (hormone therapy, multidisciplinary care, medical care, endocrinology care, surgery, psychiatric care, other, unavailable/unknown), how gender transition was begun (by themselves, not yet begun, professional support, internet groups, friends, guidance from other trans people, other, unavailable/unknown), prior use of hormones (yes), method of obtaining hormones (pharmacies without a prescription, pharmacies with a prescription, friends/colleagues, “pumpers”, SUS, internet, other), source of guidance (health professional, other travestis/transsexuals, friends, internet groups, other), prior body modification (yes), experience of gender discrimination (yes), discrimination setting (family, employment, school, public spaces, health services, religious practices, other), support at home (yes), support from other networks (yes) and name change (yes, no, unavailable/unknown).
- Characteristics of access to health services and clinical history: service sought when becoming ill (public hospital emergency service, urgent care service, primary health care center, private hospital emergency service, private clinic or consulting room, public hospital outpatient clinic, pharmacy, public specialty center/polyclinic, Family Health Strategy (FHS) professional at home, other, unavailable/unknown), accessed by the FHS (yes, no, unable to answer, unavailable/unknown), last medical consultation (in the last 12 months, 1-2 years, 2-3 years, >3 years, never been to a doctor/health service, unavailable/unknown), health insurance beneficiary (yes), preexisting condition (mental disorders, HIV, syphilis, hepatitis B, hepatitis C, other sexually transmitted infections – STIs, hypertension, obesity, asthma, diabetes, dyslipidemia, neoplasms, other, unavailable/unknown), previous use of psychotropic substances (yes), testing for STIs in the last 12 months (yes) and STI/HIV/AIDS prevention method (no prevention method most of the time, condoms, post-exposure prophylaxis, pre-exposure prophylaxis, other method, unavailable/unknown).

Age when starting attendance at the outpatient clinic (calculated by the difference between first attendance date and date of birth) underwent the Shapiro-Wilk test to assess normality [11]. Given the non-normal distribution identified, we decided to describe it using median and interquartile range values.

The “reason for seeking care”, “discrimination setting”, “service sought when becoming ill” and “preexisting illness” variable categories permitted multiple responses.

### Data source 

The data were extracted from physical and electronic medical records, based on the interdisciplinary reception interview form. Data collection was carried out by healthcare professionals at the service who were familiar with the form. The study analysis database was built using anonymized data, coded with sequential numbers to ensure confidentiality.

### Bias

The data collection instrument used was the same standardized reception interview form employed routinely by the service. During the process of its incorporation by the healthcare team, this form underwent prior revisions and adjustments. However, for the purposes of this study, it was not possible to make additional modifications to the instrument, since the data collection was based on preexisting service records.

### Study size

The analysis included 261 medical records with complete data for the period defined for attendance at the clinic and data collection.

### Statistical methods

In the initial stage of the analysis, descriptive statistics were used, presenting absolute (n) and relative (%) frequencies, stratified by gender identity. In the second stage, factors associated with the outcome “not having been accessed by the Family Health Strategy” in the period prior to starting attendance at the outpatient clinic were investigated. We opted for the joint use of prevalence ratios (PR) and odds ratios (OR) in accordance with different stages of the analysis. The PR were calculated in the bivariate analyses, while the OR were estimated in the final multivariate model, the latter being the standard measure generated by the logistic regression analysis.

Initially, bivariate analyses were performed using Pearson’s chi-square test (or Fisher’s exact test, when applicable). For each association, PRs were calculated together with their corresponding 95% confidence intervals (95%CI) and p-values. The explanatory variables were recategorized for better suitability to the statistical analysis.

In order to be included in the multivariate model, variables were selected based on the following criteria: p-value<0.20 in the bivariate analysis (for factors with potential statistical significance), PR magnitude, plausibility of other relevant variables, and consideration of possible confounding variables. Binary logistic regression (a method suitable for analyzing dichotomous outcomes) was used to identify the model that best estimated the probability of the outcome based on the selected predictors. ORs were estimated with their respective 95%CI. We adopted a 5% significance level (p-value<0.05).

The explanatory variables included: i) sociodemographic characteristics (gender identity [travestis versus others], race/skin color [mixed race and others vs. Black], schooling [incomplete high school vs. other]); ii) gender transition trajectory (how gender transition was begun [by themselves vs. other ways], method of obtaining hormones [without a prescription vs. with a prescription]); and iii) health service access conditions (last medical consultation [<12 months vs. >12 months], health insurance beneficiary [yes vs. no], seeking care at a primary health care center when becoming ill [yes vs. no]). The “how hormones are obtained” variable was excluded due to a high proportion of unavailable/ignored data (56.3%), with no impact on the sample size.

The “gender identity” and “health insurance” variables were excluded due to perfect separation [12], a condition in which the variables classify all observations correctly, which makes it impossible to estimate the logistic model, as the coefficients would tend to infinity.

The final logistic model was evaluated for discriminative ability (area under the ROC curve [receiver operating characteristic]), goodness of fit (Hosmer-Lemeshow test), and presence of multicollinearity. All analyses were performed using R software, version 4.4.3 (R Core Team, 2025). 

## Results

The sociodemographic characteristics of the sample (n=261) were presented, with a predominance of trans men (57.0%) (Table 1). Median age was 24 years (interquartile range 18-30), with 65.9% of the sample in the 18-29 age group. The majority of people were Black (61.3%), single (77.0%), heterosexual (70.1%), and had complete high school education (27.6%) or incomplete higher education (28.0%). Less than half (46.7%) reported having paid employment. Sex work was reported exclusively by trans women and travestis (5.0% of the sample); 9.2% received welfare benefits; and 44.1% lived in rented or free accommodation.

**Table 1:** Distribution of people attending the Transgender Outpatient Clinic according to sociodemographic characteristics. Brazilian Federal District, 2018-2022 (n=261)

Variables	Trans men (n=149) n (%)	Trans women (n=98) n (%)	Travestis (n=14) n (%)	Total (n=261) n (%)
**Age group **(years)				
18-29	120 (80.5)	44 (45.0)	8 (57.2)	172 (65.9)
30-39	21 (14.1)	26 (26.5)	3 (21.4)	50 (19.2)
40-49	8 (5.4)	21 (21.4)	2 (14.3)	31 (11.9)
>50	0 (0.0)	7 (7.1)	1 (7.1)	8 (3.0)
**Race/skin color**				
Mixed race	68 (45.6)	40 (40.8)	7 (50)	115 (44.1)
Black	23 (15.4)	20 (20.4)	2 (14.3)	45 (17.2)
White	49 (32.9)	34 (34.7)	4 (28.6)	87 (33.3)
Asian	2 (1.3)	2 (2.0)	1 (7.1)	5 (1.9)
Indigenous	2 (1.3)	1 (1.0)	0 (0.0)	3 (1.1)
Unavailable/unknown	5 (3.4)	1 (1.0)	0 (0.0)	6 (2.5)
**Marital status**				
Single	110 (73.8)	81 (82.7)	11 (78.6)	202 (77.4)
Married/stable union	28 (18.8)	10 (10.2)	2 (14.3)	40 (15.3)
Divorced	5 (3.4)	3 (3.1)	0 (0.0)	8 (3.1)
Widowed	0 (0.0)	1 (1.0)	1 (7.1)	2 (0.8)
Preferred not to answer	4 (2.7)	3 (3.1)	0 (0.0)	7 (2.7)
Unavailable/unknown	2 (1.3)	0 (0.0)	0 (0.0)	2 (0.8)
**Sexual orientation**				
Heterosexual	108 (72.5)	71 (72.4)	4 (28.6)	183 (70.1)
Bisexual	21 (14.1)	12 (12.2)	4 (28.6)	37 (14.2)
Homosexual	5 (3.4)	6 (6.1)	3 (21.4)	14 (5.4)
Other	15 (10.1)	9 (9.2)	3 (21.4)	27 (10.3)
Schooling				
Incomplete elementary	4 (2.7)	12 (12.2)	0 (0.0)	16 (6.1)
Complete elementary	5 (3.4)	4 (4.1)	0 (0.0)	9 (3.4)
Incomplete high school	15 (10.1)	27 (27.6)	3 (21.4)	45 (17.2)
Complete high school	46 (30.9)	23 (23.5)	3 (21.4)	72 (27.6)
Incomplete higher education	51 (34.2)	17 (17.3)	5 (35.7)	73 (28.0)
Complete higher education	22 (14.8)	10 (10.2)	2 (14.3)	34 (13.0)
Postgraduate	4 (2.7)	3 (3.1)	1 (7.1)	8 (3.1)
Unavailable/unknown	2 (1.3)	2 (2.0)	0 (0.0)	4 (1.5)
**Occupation **				
Paid activity	76 (51.0)	40 (40.8)	6 (42.9)	122 (46.7)
Sex work	0 (0.0)	11 (11.2)	2 (14.3)	13 (5.0)
Student	50 (33.6)	20 (20.4)	2 (14.3)	72 (27.6)
Unemployed	18 (12.1)	20 (20.4)	5 (35.7)	43 (16.5)
Other	6 (4.0)	5 (5.1)	1 (7.1)	12 (4.6)
Unavailable/unknown	7 (4.7)	10 (10.2)	0 (0.0)	17 (6.5)
**Welfare benefits**				
Yes	11 (7.4)	9 (9.2)	4 (28.6)	24 (9.2)
No	132 (88.6)	79 (80.6)	10 (71.4)	221 (84.7)
Unavailable/unknown	6 (4.0)	10 (10.2)	0 (0.0)	16 (6.1)
**Housing status**				
Rented or free accommodation	75 (50.4)	35 (35.7)	5 (35.7)	115 (44.1)
Own property	43 (28.9)	24 (24.5)	3 (21.4)	70 (26.8)
Reception/shelter institution	1 (0.7)	5 (5.1)	0 (0.0)	6 (2.3)
Street dweller	0 (0.0)	2 (2.0)	2 (14.3)	4 (1.5)
Unavailable/unknown	30 (20.1)	32 (32.7)	4 (28.6)	66 (25.3)

39.1% of participants found out about the clinic through friends (Table 2). The main reasons for seeking care were hormone therapy (76.6%) and multidisciplinary care (73.6%). Before consulting, 56.7% were already using hormones – 37.8% without a prescription (including 6 of the 10 travestis) and 35.2% with a prescription (especially trans men). Body modifications had already been performed by 48.0% (59.2% trans women). Gender discrimination affected 73.2% of the sample, and 25.3% had legally changed their legal name (no *travestis *had done so).

**Table 2:** Distribution of people attending the Transgender Outpatient Clinic according to characteristics of their gender transition trajectory. Brazilian Federal District, 2018-2022 (n=261)

Variables	Trans men (n=149) n (%)	Trans women (n=98) n (%)	Travestis (n=14) n (%)	Total (n=261)
**Prior knowledge about the Transgender Clinic**				
From friends or acquaintances	69 (46.3)	27 (27.6)	6 (42.9)	102 (39.1)
Referred by health service	25 (16.8)	24 (24.5)	1 (7.1)	50 (19.2)
Social networks	24 (16.1)	18 (18.4)	5 (35.7)	47 (18)
Social assistance referral center or Specialized social assistance referral center	15 (10.1)	15 (15.3)	1 (7.1)	31 (11.9)
Outros	10 (6.7)	12 (12.2)	1 (7.1)	23 (8.8)
Unavailable/unknown	8 (5.4)	4 (4.1)	0 (0.0)	12 (4.6)
**Reason for seeking care**				
Hormone therapy	118 (79.2)	70 (71.4)	12 (85.7)	200 (76.6)
Multi-professional care	114 (76.5)	70 (71.4)	8 (57.1)	192 (73.6)
Medical care	74 (49.7)	52 (53.1)	7 (50)	133 (51.0)
Endocrinology care	52 (34.9)	35 (35.7)	4 (28.6)	91 (34.9)
Surgery	47 (31.5)	38 (38.8)	5 (35.7)	90 (34.5)
Psychiatric care	42 (28.2)	23 (23.5)	1 (7.1)	66 (25.3)
Other	6 (4.0)	5 (5.1)	1 (7.1)	12 (4.6)
Unavailable/unknown	0 (0.0)	1 (1.0)	0 (0)	1 (0.4)
**How gender transition was begun**				
By themselves	42 (28.2)	48 (49.0)	7 (50.0)	97 (37.2)
Not started yet	68 (45.6)	22 (22.4)	3 (21.4)	93 (35.6)
Professional care	29 (19.5)	17 (17.3)	2 (14.3)	48 (18.4)
Internet groups	7 (4.7)	5 (5.1)	0 (0.0)	12 (4.6)
Friends	7 (4.7)	2 (2.0)	1 (7.1)	10 (3.8)
Unavailable/unknown	5 (3.4)	8 (8.2)	1 (7.1)	14 (5.4)
Prior hormone use	71 (47.7)	67 (68.4)	10 (71.4)	148 (56.7)
**How hormones are obtained**				
Pharmacy without a prescription	19 (26.8)	31 (46.3)	6 (60.0)	56 (37.8)
Pharmacy with a prescription	25 (35.2)	16 (23.9)	1 (10.0)	42 (28.4)
Internet	9 (12.7)	2 (3.0)	0 (0.0)	11 (7.4)
Friends/work colleagues	5 (7.0)	0 (0.0)	0 (0.0)	5 (3.4)
Industrial silicone pumpers	0 (0.0)	3 (4.5)	0 (0.0)	3 (2.0)
Brazilian Unified Health System	0 (0.0)	1 (1.5)	0 (0.0)	1 (0.7)
Other	1 (1.4)	2 (3)	0 (0.0)	3 (2.0)
Unavailable/unknown	12 (16.9)	12 (17.9)	3 (30.0)	27 (18.2)
**Source of guidance **				
Health professional	23 (32.4)	17 (25.4)	1 (10.0)	41 (27.7)
Other travestis/trans	2 (2.8)	12 (17.9)	2 (20.0)	16 (10.8)
Internet groups	6 (8.5)	5 (7.5)	0 (0.0)	11 (7.4)
Friends	6 (8.5)	2 (3.0)	1 (10.0)	9 (6.1)
Other	1 (1.4)	2 (3.0)	0 (0.0)	3 (2.0)
Unavailable/unknown	33 (46.5)	29 (43.3)	6 (60.0)	68 (45.9)
Prior body modification	60 (40.3)	58 (59.2)	7 (50.0)	125 (47.9)
Experience of gender discrimination	110 (73.8)	68 (69.4)	13 (92.9)	191 (73.2)
**Discrimination setting**				
Family	99 (66.4)	54 (55.1)	12 (85.7)	165 (63.2)
Public spaces	56 (37.6)	48 (49.0)	12 (85.7)	116 (44.4)
Employment	23 (15.4)	23 (23.5)	3 (21.4)	49 (18.8)
School	18 (12.1)	14 (14.3)	2 (14.3)	34 (13.0)
Religious practices	11 (7.4)	1 (1.0)	0 (0.0)	12 (4.6)
Health services	2 (1.3)	3 (3.1)	0 (0.0)	5 (1.9)
Other	2 (1.3)	6 (6.1)	0 (0.0)	8 (3.1)
Support at home	90 (60.4)	33 (33.7)	3 (21.4)	126 (48.3)
Support from other networks	66 (44.3)	26 (26.5)	7 (50.0)	99 (37.9)
**Name change **				
Yes	34 (22.8)	32 (32.7)	0 (0.0)	66 (25.3)
No	114 (76.5)	66 (67.3)	14 (100.0)	194 (74.3)
Unavailable/unknown	1 (0.7)	0 (0.0)	0 (0.0)	1 (0.4)

There was predominance of use of urgent/emergency services (56.7% in total) compared to primary health care centers (21.0%); 69.0% had not been accessed by the FHS; and 17.2% had health insurance (Table 3). High prevalence rates of reported mental disorders (46.4%) and psychotropic sustance use (49.0%) were observed, with alcohol, tobacco and marijuana being the most frequently cited. HIV infection was reported by 9.8% among trans women and travestis, with no cases among trans men. The proportion of people who reported not using HIV and other STI prevention methods most of the time was noteworthy, especially among trans men (37.6%). Use of post-exposure prophylaxis and pre-exposure prophylaxis was low (5.1%) and was restricted to trans women.

**Table 3:** Distribution of people attending the Transgender Outpatient Clinic according to characteristics of access to health services and clinical history. Brazilian Federal District, 2018-2022 (n=261)

Variable	Trans men (n=149) n (%)	Trans women (n=98) n (%)	Travestis (n=14) n (%)	Total (n=261)
**Service sought when becoming ill**				
Public hospital emergency department	48 (32.2)	26 (26.5)	5 (35.7)	79 (30.3)
Urgent care service	43 (28.9)	25 (25.5)	1 (7.1)	69 (26.4)
Primary care center	32 (21.5)	21 (21.4)	2 (14.3)	55 (21.1)
Private hospital emergency department	20 (13.4)	17 (17.3)	5 (35.7)	42 (16.1)
Private outpatient clinic or consulting room	21 (14.1)	17 (17.3)	3 (21.4)	41 (15.7)
Public hospital outpatient clinic	10 (6.7)	15 (15.3)	0 (0.0)	25 (9.6)
Pharmacy	11 (7.4)	6 (6.1)	1 (7.1)	18 (6.9)
Public specialty center or polyclinic	4 (2.7)	9 (9.2)	0 (0.0)	13 (5.0)
Family health team professional at home	0 (0.0)	0 (0.0)	0 (0.0)	0 (0.0)
Other	9 (6.0)	5 (5.1)	3 (21.4)	17 (6.5)
Unavailable/unknown	14 (9.4)	16 (16.3)	1 (7.1)	31 (11.9)
**Service accessed via the family health strategy **				
Yes	15 (10.1)	9 (9.2)	0 (0.0)	24 (9.2)
No	99 (66.4)	68 (69.4)	13 (92.9)	180 (69.0)
Unable to answer	10 (6.7)	4 (4.1)	0 (0.0)	14 (5.3)
Unavailable/unknown	25 (16.8)	17 (17.3)	1 (7.1)	43 (16.5)
**Last medical consultation **				
In the last 12 months	102 (68.5)	68 (69.4)	8 (57.2)	178 (68.2)
1-2 years	11 (7.4)	10 (10.2)	2 (14.3)	23 (8.8)
2-3 years	12 (8.0)	5 (5.1)	1 (7.1)	18 (6.9)
>3 years	11 (7.4)	4 (4.1)	2 (14.3)	17 (6.5)
Never been to the doctor or to a health service	1 (0.7)	1 (1.0)	0 (0.0)	2 (0.8)
Unavailable/unknown	12 (8.0)	10 (10.2)	1 (7.1)	23 (8.8)
Health insurance beneficiary	22 (14.8)	20 (20.4)	3 (21.4)	45 (17.2)
**Preexisting illness **				
Mental disorders	72 (48.3)	45 (45.9)	4 (28.6)	121 (46.4)
Hypertension	3 (2.0)	8 (8.2)	1 (7.1)	12 (4.6)
HIV	0 (0.0)	9 (9.2)	2 (14.3)	11 (4.2)
Obesity	3 (2.0)	4 (4.1)	0 (0.0)	7 (2.7)
Syphilis	1 (0.7)	4 (4.1)	1 (7.1)	6 (2.3)
Asthma	2 (1.3)	1 (1.0)	0 (0.0)	3 (1.1)
Diabetes	0 (0.0)	1 (1.0)	1 (7.1)	2 (0.8)
Dyslipidemia	1 (0.7)	1 (1.0)	0 (0.0)	2 (0.8)
Neoplasms	0 (0.0)	1 (1.0)	0 (0.0)	1 (0.4)
Hepatitis C	0 (0.0)	1 (1.0)	0 (0.0)	1 (0.4)
Other sexually transmitted infection (STI)	0 (0.0)	1 (1.0)	0 (0.0)	1 (0.4)
Hepatitis B	0 (0.0)	0 (0.0)	0 (0.0)	0 (0.0)
Other	69 (46.3)	34 (34.7)	4 (28.6)	107 (41.0)
Unavailable/unknown	17 (11.4)	11 (11.2)	3 (21.4)	31 (11.9)
**Prior psychotropic substance use **	73 (49.0)	47 (48.0)	8 (57.1)	128 (49.0)
**Tested for STI in the last 12 months**	48 (32.2)	54 (55.1)	9 (64.3)	111 (42.5)
**STI/HIV/AIDS prevention method**				
No prevention method most of the time	56 (37.6)	6 (6.1)	1 (7.1)	63 (24.1)
Condom	8 (5.4)	41 (41.8)	6 (42.9)	55 (21.1)
Pre-exposure prophylaxis	0 (0.0)	5 (5.1)	0 (0.0)	5 (1.9)
Post-exposure analysis	0 (0.0)	5 (5.1)	0 (0.0)	5 (1.9)
Other method	15 (10.1)	13 (13.3)	2 (14.3)	30 (11.5)
Unavailable/unknown	70 (47.0)	39 (39.8)	5 (35.7)	114 (43.7)

Not seeking care at a primary health care center was significantly associated with higher probability of not being accessed by the FHS (PR 1.36; 95%CI 1.11; 1.66; p-value<0.001) (Table 4).

**Table 4:** Unadjusted prevalence ratios (PR) and 95% confidence intervals (95%CI) for not being accessed by the family health strategy (FHS) among people attending the Transgender Outpatient Clinic, according to sociodemographic, gender transition trajectory and health service access conditions characteristics. Brazilian Federal District, 2018-2022 (n=261)

Variable	Not accessed by the FHS n (%)	PR (95CI%)	p-value
Sociodemographic			
Gender			0.371^a^
Travestis	13 (100.0)	1.14 (1.08; 1.21)	
Other	167 (87.4)	1.00	
**Age group **(years)			0.546^a^
18-39	152 (87.4)	0.94 (0.84; 1.05)	
>40	28 (93.3)	1.00	
**Race/skin color**			0.052^a^
Mixed race, White and other	149 (90.3)	1.15 (0.97; 1.37)	
Black	29 (78.4)	1.00	
**Sexual orientation**			0.270^b^
Heterosexual	118 (86.1)	0.93 (0.85; 1.02)	
Other	62 (92.5)	1.00	
**Schooling **			0.053^a^
Incomplete high school education	38 (97.4)	1.13 (1.05; 1.23)	
Other	140 (85.9)	1.00	
**Gender transition trajectory **			
**How gender transition was begun **			0.270^b^
By themselves	78 (91.8)	1.07 (0.97; 1.18)	
Others	102 (85.7)	1.00	
**Prior hormone use **			0.630^b^
No	71 (86.6)	0.96 (0.87; 1.07)	
Yes	106 (89.8)	1.00	
**How hormones are obtained **			0.124^a^
Without a prescription	54 (94.7)	1.13 (0.96; 1.33)	
With a prescription	26 (83.9)	1.00	
**Support from other networks **			0.449^a^
No	60 (90.9)	1.06 (0.95; 1.19)	
Yes	71 (85.5)	1.00	
**Health service access conditions **			
**Last medical consultation **			0.109^b^
Last 12 months	122 (85.3)	0.90 (0.82; 0.99)	
Other	55 (94.8)	1.00	
**Health insurance beneficiary**			0.052^a^
Yes	38 (97.4)	1.13 (1.04; 1.23)	
No	131 (86.2)	1.00	
**Seeks care at a primary care center when becoming ill**			<0.001^b^
No	149 (93.7)	1.36 (1.11; 1.66)	
Yes	31 (68.9)	1.00	
**Preexisting hypertension**			0.611^a^
Yes	10 (100.0)	1.14 (1.08; 1.2)	
No	170 (87.6)	1.00	

^a^Fisher’s exact test; ^b^Pearson’s chi-square test.

The adjusted logistic model demonstrated good discriminative capacity (ROC 0.847) and goodness of fit and calibration, confirmed by the Hosmer-Lemeshow test (p-value 0.963); absence of multicollinearity (variance inflation factor test 1); analysis of outliers; and adequate distribution of residuals. Seeking care at a primary health care center reduced the odds of absence of linkage to the FHS by 86.0% (OR 0.14; 95%CI 0.05; 0.37; p-value<0.001) (Table 5). The high intercept (OR 25.27; 95%CI 2.39; 266.77; p-value 0.015) indicated a high baseline probability of not having been accessed by the FHS. Other variables did not show statistical significance in Tables 4 e 5.

**Table 5:** Adjusted odds ratios (OR) and 95% confidence intervals (95%CI) for not being accessed by the family health strategy among people attending the Transgender Outpatient Clinic, according to sociodemographic, gender transition trajectory and health service access conditions characteristics. Brazilian Federal District, 2018-2022 (n=261)

Variables	Estimated coefficient (β)	Standard error	p-value	OR (95%CI)
Intercept	3.23	1.20	0.015	25.27 (2.39; 266.77)
**Mixed race and others (versus Black)**	0.94	0.55	0.094	2.55 (0.87; 7.45)
**Level of schooling other than incomplete high school education**	-1.86	1.07	0.086	0.16 (0.02; 1.26)
**Gender transition begun by themselves ** (**vs. other forms)**	0.68	0.52	0.194	1.98 (0.71; 5.50)
**Last medical consultation >12 months ** (**vs. <12 months)**	0.82	0.68	0.235	2.28 (0.60; 8.63)
**Seeks care at a primary health center when becoming ill **(vs. no)	-1.95	0.49	<0.001	0.14 (0.05; 0.37)

## Discussion

The results revealed that more than half of the people attending the Federal District Transgender Outpatient Clinic were trans men, followed by trans women and travestis, predominantly young, Black, single, heterosexual individuals with at least high school education. The main challenges identified included a low rate of identity document updating for name and gender change, diverse discrimination settings, starting gender transition without professional support, high prevalence of mental disorders, and concentration of care in emergency services instead of PHC. Prior attendance at primary health care centers increased linkage with the FHS, which reduced the likelihood of lack of care.

Disparities were also evident between the gender categories studied, especially among travestis. Despite constituting the smallest percentage of the total, they presented the most precarious socioeconomic conditions. This reality was confirmed by a study conducted in Rio de Janeiro which, despite methodological differences, also identified travestis as the group with the poorest indicators for income, education and access to health [13].

In Brazil, lack of official data on gender identity makes it difficult to accurately characterize this population. Available estimates suggest that 1% of the adult population in 2021 identified as binary trans people, with a balanced distribution between men and women [14]. However, these estimates do not distinguish between the trans women and travesti categories.

In comparison with national data, overrepresentation of young people was identified in this sample (more than twice that of the general Brazilian population), while the proportion of Black people approached the national composition, representing more than half of the total [15]. The possible intersectionality of these variables intensifies health disparities, as in the case of young Black trans people in Canada, who, in 2023, were twice as likely to not receive necessary physical care compared to their White peers [16].

The proportion of trans people with complete higher education in this study was 23% lower than that of the general population aged 25 or older [15]. School exclusion and structural barriers in the academic trajectory of trans people lead to educational dropout, limit professional opportunities and perpetuate socioeconomic marginalization in adulthood [17].

The sample investigated presented a lower percentage of heterosexuals when compared to the general population [18]. Gender identity and sexual orientation are distinct, non-fixed dimensions and can vary throughout life [19]. Both trans and cisgender (cis) people can have any sexual orientation, which shows the plurality of human identity experiences.

The high frequency of gender identity-based discrimination was confirmed by research conducted in five Brazilian capitals with trans women and travestis [20]. The increase in this form of discrimination was associated with a history of violence and sociodemographic characteristics comprising greater vulnerability. 

The greater demand for hormone therapy and multidisciplinary care when compared to surgery can be understood by taking the principle of gender self-determination, which stems from identity processes built from life experiences. This includes hormone therapy as an essential resource for gender affirmation, regardless of the need for surgical procedures [21].

The practice of hormone self-medication in this population has already been recorded in Brazilian research [8]. The predominance of starting gender transition without professional supervision (especially among trans women and travestis) contrasts with findings from a Chilean study [22], in which 58% of trans people using hormones stated that they obtained them through health professionals. The regional inequality in the provision of specialized services in Brazil [10] hinders this population’s access to adequate care. In Brazil, the fact that testosterone is a controlled medication may restrict hormone therapy without professional supervision among trans men, since this procedure constitutes one of the main demands for body modification in this group [23].

Lack of legal name change negatively impacts socioeconomic indicators and leads people to postpone seeking health services [24]. This structural and institutional violence, when intersecting with other forms of oppression, constitutes a barrier to diverse social rights. In Argentina [25], trans women discriminated against by health professionals were three times more likely to avoid health services than those not discriminated against. 

Low health insurance coverage – lower than the average for the Federal District (33%) and lower than the national average (25%) [26] – indicated that most of the people investigated are SUS users. Despite the increase in FHS coverage in the Federal District from 34% in 2017 to 77% in 2023 [27], this expansion proved insufficient to overcome the care gaps verified in this study, within the scope of PHC.

Regarding preexisting health conditions, some similarities were observed with North American research, in which trans women reported poorer health outcomes compared to trans men, with the exception of depression [28]. Although the present research did not specify types of mental disorders, prevalence of self-reported cases of these conditions was four times higher than prevalence of positive screening for depression in the Brazilian adult population [29]. Beyond gender-affirming care, an approach that fully incorporates mental health is needed, addressing discrimination in both individual and social contexts [30].

Use of psychotropic substances by trans women and travestis showed a similar percentage to that found in previous Brazilian research [31], which pointed to alcohol, tobacco and marijuana as the most consumed substances. Multiple use of substances, when associated with greater violence, unemployment and informal work, is possibly a strategy for coping with suffering and marginalization.

Low adherence to pre-exposure prophylaxis among trans women is corroborated by findings from a national study [32], which shows that only 6% of trans people in Brazil use this prophylaxis (vs. 94% of cis people). HIV and other STI prevention requires integrated approaches that combine diverse strategies and specific adaptations for each gender group studied.

This research presented limitations, mainly due to its cross-sectional design, which prevents inference of causal or temporal relationships. Furthermore, self-reporting can compromise the reliability of sensitive variables and introduce information biases.

The non-probabilistic sample only included trans people and travestis treated at the clinic, which constitutes a selection bias by not incorporating those who had not yet accessed the service. Non-binary individuals and those of other gender identities, although treated by the service, were also not included in the study. However, an attempt was made to characterize in detail the other groups that had access to the service.

The context of the Federal District, with its particular service structure, may also differ from other realities and limit generalization of the results. Although the outpatient clinic recorded data not captured by other services, the fact of this research being limited to routinely recorded variables may not encompass important social and cultural aspects. High proportions of unavailable or unknown data for some variables, such as individual and family income, had to be excluded because they restricted the analysis. The modest sample size may have been insufficient to detect significant differences between the groups, although it allowed for descriptive and associational analyses.

Furthermore, significant underrepresentation of travestis and overrepresentation of trans men were observed, limiting specific analyses for these groups. The stratified presentation of data on travestis was maintained as a methodological and political strategy to highlight their specific identity and confront their historical social invisibility, even though the term “trans” is frequently used in the literature in its broad sense to encompass diverse gender identities. Furthermore, our investigation did not analyze whether broader demands of the people cared for were met, nor did it investigate in detail their connection with services provided by the care network and other health needs. 

Despite its limitations, this study represents a relevant contribution, serving as an exploratory basis for future research, including qualitative research.

In conclusion, the results showed sociodemographic inequities, inequalities in gender transition trajectories and access to health services, with underutilization of PHC. Linkage with primary health care centers proved to be protective, emphasizing the FHS as a model for comprehensive care. In addition to linking PHC with specialized services, it is necessary to expand, equitably, FHS coverage in the Federal District to reduce the demand for urgent care services and implement preventive measures for mental disorders and other morbidities.

The findings also revealed the need for intersectoral policies at the Federal District and national levels to ensure full social rights. These actions, combined with anti-discrimination campaigns, are essential for mitigating barriers and guaranteeing equity in access, in keeping with the principles of the SUS. 

## Data Availability

In order to protect study participant privacy and ensure confidentiality, only consolidated data is available. The complete database remains restricted due to the sensitive and individualized nature of the information, which, even after anonymization, may present risks of identification.
