# Supplementary material for: Sociodemographic profile and access to health services among people attending the Transgender Outpatient Clinic: a cross-sectional study, Brazilian Federal District, 2018-2022
Source: Epidemiol Serv Saude. 2026 Aug 3;35:e20251037. doi: 10.1590/S2237-96222026v35e20251037.en (PMC13686418; doi:10.1590/S2237-96222026v35e20251037.en)
Supplement: Translation

# Translation: Perfil sociodemográfico e acesso a serviços de saúde entre pessoas atendidas no Ambulatório Trans: estudo transversal, Distrito Federal, 2018-2022

**DOI:** 10.1590/S2237-96222026v35e20251037.pt

**Authors:** Francisca Lidiane Sampaio Freitas, Ximena Pamela Díaz Bermúdez, Edgar Merchán-Hamann

**Affiliations:** 1 Universidade de Brasília Faculdade de Ciências da Saúde Brasília DF Brasil Universidade de Brasília, Faculdade de Ciências da Saúde, Brasília, DF, Brasil

**Keywords:** Pessoas Transgênero, Travestis, Acesso a Serviços de Saúde, Atenção Primária à Saúde, Estudos Transversais

## Abstract

**Objetivos:**

Caracterizar perfil sociodemográfico, percurso de transição de gênero e condições de acesso a serviços de saúde de pessoas trans e travestis, além de identificar fatores associados a não ter sido acessado(a) pela estratégia saúde da família (ESF).

**Métodos:**

Estudo transversal mediante análise de prontuários de pessoas trans e travestis (≥18 anos) atendidas no Ambulatório Trans do Distrito Federal, 2018-2022. Realizou-se análise descritiva estratificada por gênero. Os fatores associados foram analisados por meio de análise bivariada (razão de prevalência [RP] com intervalo de confiança de 95% [IC95%]) e análise multivariada por regressão logística binária (odds ratio [OR] ajustado com IC95%).

**Resultados:**

Entre 261 prontuários (57,0% homens trans, 37,6% mulheres trans e 5,4% travestis), predominaram pessoas jovens (66,0%, 18-29 anos), negras (61,3%), com ocupação remunerada (46,7%) e ensino médio completo ou menor nível (54,3%); 25,0% retificaram o nome, 73,2% relataram discriminação, e 49,0% das mulheres trans e travestis iniciaram a transição sem acompanhamento. Doenças psiquiátricas (46,4%) foram as mais relatadas; e 17,2% tinham plano de saúde. Observou-se maior utilização de serviços públicos de urgência e emergência (56,7%) que na atenção primária (21,1%). O fato de buscar atendimento na unidade básica de saúde (UBS) quando do adoecimento diminuiu em 86,0% a chance do desfecho negativo (OR 0,14; IC95% 0,05; 0,37).

**Conclusão:**

Evidenciaram-se iniquidades sociodemográficas, desigualdades na trajetória de transição de gênero e no acesso à saúde, com subutilização da atenção primária. O vínculo com a UBS mostrou-se fator protetor, o que reforça a ESF como essencial ao cuidado integral.

**Aspectos éticos**

A presente pesquisa respeitou os princípios éticos, obtendo os seguintes dados de aprovação:

**Comitê de ética em pesquisa** **Número do parecer**, **Certificado de apresentação de apreciação ética**, **Data de aprovação**

Universidade de Brasília 6.333.242, 69644223.4.0000.0030, 29/9/2023

Secretaria de Saúde do Distrito Federal 6.650.598, 69644223.4.3002.5553, 15/2/2024

**Registro do consentimento livre e esclarecido** Dispensado.

## Introdução

O Sistema Único de Saúde (SUS) alcançou avanços significativos na assistência à população brasileira. Entretanto, persistem desafios político-programáticos, sociais e econômicos que impedem sua plena efetivação [1]. O acesso à saúde não se limita a atendimentos pontuais, mas constitui um processo contínuo de cuidado, condicionado à efetividade das ações assistenciais [2]. A integralidade em saúde requer que os serviços, em todos os níveis de atenção, organizem processos e práticas para acolher demandas de modo adequado [3]. 

Os preceitos da Política Nacional de Atenção Básica [4] definem a Atenção Primária à Saúde (APS) como porta de entrada preferencial do SUS, a qual deve ser livre de qualquer forma de exclusão. A estratégia saúde da família (ESF) é um modelo assistencial da APS, com foco na prevenção, promoção e recuperação da saúde. Cabe aos estados e ao Distrito Federal fortalecê-la como ação prioritária, a partir da atuação em territórios definidos. Ter sido acessado(a) pela ESF indica o acompanhamento de uma situação de saúde ou a realização de visita domiciliar pela equipe, por exemplo. Isso pode reduzir a procura por emergências, prevenir hospitalizações desnecessárias e garantir os encaminhamentos para a atenção especializada.

Na prática, constitui um desafio assegurar o acesso de pessoas transgênero (trans) aos serviços da APS e da atenção especializada. Essa população enfrenta uma realidade marcada por múltiplas barreiras e violações sistemáticas de direitos, especialmente quando suas necessidades em saúde exigem cuidado integral que transcende o processo de afirmação de gênero [5]. 

No Distrito Federal, 1% das pessoas adultas se autodeclarou trans em 2021 [6]. A realidade dessa população é marcada por uma contradição: apesar da existência do Ambulatório Trans, que proporciona cuidado especializado com abordagem clínica e psicossocial, ainda subsistem problemas estruturais, como a fragmentação da rede assistencial, a sobrecarga do serviço e a capacidade limitada de acolhimento pela APS [7]. 

Este estudo mostrou-se relevante ao preencher lacunas na investigação dos percursos assistenciais (com destaque para a APS), além de contribuir para embasar políticas de saúde mais equitativas para pessoas trans e travestis em todos os níveis de cuidado. Foram identificados dois artigos científicos [8,9] produzidos no contexto do Ambulatório Trans, mas que abordam temáticas distintas desta pesquisa. 

Este estudo teve como objetivos: caracterizar o perfil sociodemográfico, o percurso de transição de gênero e as condições de acesso a serviços de saúde de travestis, mulheres trans e homens trans; e identificar fatores associados a não ter sido acessado(a) pela ESF.

## Métodos

### Delineamento do estudo

Trata-se de estudo transversal realizado com dados da pesquisa denominada “Projeto Athena: construção de evidências para a ação e tomada de decisão em saúde integral, doenças infecciosas e coinfecções”. Esse projeto abrange uma parceria entre a Universidade de Brasília e o Centro Especializado em Doenças Infecciosas da Secretaria de Estado de Saúde do Distrito Federal.

### Contexto

Este estudo foi realizado no Ambulatório Trans, unidade vinculada ao Centro Especializado em Doenças Infecciosas, criado em 2017 [7]. No entanto, a habilitação do Ambulatório pelo Ministério da Saúde para atendimento especializado só ocorreu em agosto de 2024 [10].

As principais formas de acesso ao serviço são: demanda espontânea; encaminhamento pelo centro de referência especializado de assistência social (Creas) da diversidade; e referência por outros serviços da rede. O acolhimento realizado pela equipe multiprofissional segue três etapas: grupo de admissão; entrevista individual, por meio do formulário de acolhimento; e agendamento das consultas conforme as necessidades [7]. 

### Participantes do estudo

A amostra por conveniência foi constituída de prontuários consecutivos de pessoas trans e travestis, com 18 anos ou mais, que ingressaram no ambulatório no período 2018-2022. O ano de 2020 foi excluído devido ao impacto disruptivo da pandemia de covid-19 e a interrupção de serviços no ambulatório. Tal exclusão buscou garantir que a análise reflita a dinâmica regular de acesso aos serviços, sem a distorção causada por esse evento excepcional.

### Variáveis 

Para garantir a robustez estatística e preservar o tamanho amostral adequado para as análises de associação, realizou-se a recategorização de variáveis mediante dois critérios principais: agrupamento de categorias com frequência insuficiente e preservação do poder estatístico nas análises. Essa abordagem resultou em uma distribuição organizada em três blocos distintos, conforme a seguir.

- Características sociodemográficas: faixa etária da admissão (18-29, 30-39, 40-49, 50+ anos), orientação sexual (heterossexual, homossexual, bissexual, outro), raça/cor da pele (parda, preta, branca, amarela, indígena, não sabe), estado civil (solteiro/a, casado/a, união estável, divorciado/a, viúvo/a, não quis responder, indisponível/ignorado), escolaridade (fundamental incompleto, fundamental completo, médio incompleto, médio completo, superior incompleto, superior completo, pós-graduação, indisponível/ignorado), ocupação (atividade remunerada, comércio sexual, desempregado/a, estudante, outro, indisponível/ignorado), benefícios assistenciais (sim, não, indisponível/ignorado) e situação de moradia (instituição de acolhimento/abrigo, situação de rua, imóvel alugado ou cedido, imóvel próprio, indisponível/ignorado).
- Características do percurso da transição de gênero: conhecimento prévio do Ambulatório Trans (por amigos ou conhecidos, encaminhamento por serviço de saúde, redes sociais, centro de referência de assistência social – Cras e/ou Creas, outros, indisponível/ignorado), motivação para busca de atendimento (hormonização, acompanhamento multiprofissional, acompanhamento médico, acompanhamento endocrinológico, cirurgias, acompanhamento psiquiátrico, outros, indisponível/ignorado), modo de início da transição de gênero (por conta própria, ainda não iniciada, acompanhamento profissional, grupos da internet, amigos, orientação de outras pessoas trans, outros, indisponível/ignorado), uso prévio de hormônios (sim), forma de obtenção dos hormônios (farmácias sem receita médica, farmácias com receita médica, amigos/colegas de trabalho, “bombadeiras”, SUS, internet, outros), fonte das orientações (profissional de saúde, outras travestis/transexuais, amigos, grupos da internet, outros), modificação corporal prévia (sim), experiência de discriminação por gênero (sim), contexto da discriminação (família, emprego, escola, espaços públicos, serviços de saúde, práticas religiosas, outros), apoio intradomiciliar (sim), apoio de outras redes (sim) e retificação do nome (sim, não, indisponível/ignorado).
- Características de condições de acesso a serviços de saúde e antecedentes clínicos: serviço procurado ao adoecer (emergência de hospital público, unidade de pronto atendimento – UPA, unidade básica de saúde – UBS, emergência de hospital privado, ambulatório ou consultório de clínica privada, ambulatório de hospital público, farmácia, centro de especialidades/policlínica pública, profissional da ESF em casa, outro, indisponível/ignorado), acessado(a) pela ESF (sim, não, não sabe, indisponível/ignorado), última consulta médica (nos últimos 12 meses, 1-2 anos, 2-3 anos, >3 anos, nunca foi a médico/serviço de saúde, indisponível/ignorado), beneficiário(a) de plano de saúde (sim), doença preexistente (doenças psiquiátricas, HIV, sífilis, hepatite B, hepatite C, outras infecções sexualmente transmissíveis – ISTs, hipertensão arterial, obesidade, asma, diabetes, dislipidemia, neoplasias, outras, indisponível/ignorado), uso prévio de substâncias psicoativas (sim), testagem para ISTs nos últimos 12 meses (sim) e forma de prevenção de ISTs/HIV/aids (não se previne a maior parte das vezes, preservativo, profilaxia pós-exposição, profilaxia pré-exposição, outro método, indisponível/ignorado).

A idade de admissão (calculada pela diferença entre a data de ingresso e a data de nascimento) foi submetida ao teste de Shapiro-Wilk para a avaliação de normalidade [11]. Dada a distribuição não normal identificada, optou-se por descrevê-la com mediana e intervalo interquartílico.

As categorias das variáveis “motivação para busca de atendimento”, “contexto de discriminação”, “serviço procurado ao adoecer” e “doença preexistente” admitiram resposta múltipla. 

### Fonte de dados 

Os dados foram extraídos de prontuários físicos e eletrônicos, com base no formulário da entrevista interdisciplinar de acolhimento. A coleta foi realizada por profissionais de saúde do serviço habituados ao manejo do instrumento. O banco de análise do estudo foi constituído a partir de dados anonimizados, codificados com números sequenciais para garantir a confidencialidade.

### Viés 

O instrumento de coleta de dados utilizado correspondeu ao mesmo formulário padronizado empregado na rotina do serviço. Durante seu processo de incorporação pela equipe de saúde, esse formulário passou por revisões e ajustes prévios. Entretanto, para fins deste estudo, não foi possível realizar modificações adicionais ao instrumento, uma vez que a coleta de dados se baseou em registros preexistentes do serviço.

### Tamanho do estudo

A análise incluiu 261 prontuários com dados completos para o período definido de admissão e coleta.

### Métodos estatísticos

Na etapa inicial da análise, utilizou-se estatística descritiva com apresentação de frequências absolutas (n) e relativas (%), estratificadas por identidade de gênero. Na segunda etapa, foram investigados fatores associados ao desfecho “não ter sido acessado(a) pela ESF” no período anterior à admissão no ambulatório. Optou-se pelo uso conjunto da razão de prevalência (RP) e da razão de chances (*odds ratio*, OR) para atender a diferentes etapas da análise. A RP foi calculada nas análises bivariadas, enquanto a OR foi estimada no modelo multivariado final, sendo esta última a medida padrão gerada pela regressão logística. 

Inicialmente, foram realizadas análises bivariadas com testes qui-quadrado de Pearson (ou teste exato de Fischer, quando aplicável). Para cada associação, calcularam-se as RP com seus intervalos de confiança de 95% (IC95%) e p-valor correspondentes. As variáveis explicativas foram recategorizadas para melhor adequação à análise estatística.

Para inclusão no modelo multivariado, as variáveis foram selecionadas com base nos seguintes critérios: o p-valor<0,20 na análise bivariada (para fatores com potencial de significância estatística), a magnitude da RP, a plausibilidade de outras variáveis relevantes e a consideração de possíveis variáveis de confusão. Utilizou-se regressão logística binária (método adequado à análise de desfechos dicotômicos) para identificar o modelo que melhor estima a probabilidade do desfecho com base nos preditores selecionados. Foram estimadas as razões de chances (*odds ratio*, OR) com seus respectivos IC95%. O nível de significância adotado foi de 5% (p-valor<0,05).

As variáveis explicativas incluíram: i) características sociodemográficas (identidade de gênero [travestis versus outros], raça/cor da pele [pardos e outros vs. pretos], escolaridade [ensino médio incompleto vs. outras]); ii) percurso da transição de gênero (modo de início da transição de gênero [por conta própria vs. outras formas], forma de obtenção de hormônio [sem receita médica vs. com receita médica]); e iii) condições de acesso a serviços de saúde (última consulta médica [<12 meses vs. >12 meses], beneficiário(a) de plano de saúde [sim vs. não], busca por atendimento na UBS quando adoece [sim vs. não]). A variável “como conseguiu hormônio” foi excluída por conter alta proporção de dados indisponíveis/ignorados (56,3%), sem impacto sobre o tamanho da amostra. 

As variáveis “identidade de gênero” e “plano de saúde” foram excluídas devido à separação perfeita [12], condição em que as variáveis classificam todas as observações corretamente, o que impossibilita a estimação do modelo logístico, pois os coeficientes tenderiam ao infinito.

O modelo logístico final foi avaliado quanto à capacidade discriminativa (*área sob a curva ROC *[*receiver operating characteristic *– característica de operação do receptor]), à adequação do ajuste (teste de Hosmer-Lemeshow) e à presença de multicolinearidade. Todas as análises foram realizadas no software R, versão 4.4.3 (R Core Team, 2025). 

## Resultados

Foram apresentadas as características sociodemográficas da amostra (n=261), com a predominância de homens trans (57,0%) (Tabela 1). A mediana de idade foi de 24 anos (intervalo interquartil 18-30), com 65,9% da amostra na faixa etária 18-29 anos. A maioria das pessoas era negra (61,3%), solteira (77,0%), heterossexual (70,1%) e tinha ensino médio completo (27,6%) ou superior incompleto (28,0%). Menos da metade (46,7%) referiu possuir atividade remunerada. O comércio sexual foi relatado exclusivamente por mulheres trans e travestis (5,0% da amostra); 9,2% recebiam benefícios sociais; e 44,1% residiam em imóveis alugados ou cedidos. 

**Tabela 1:** Distribuição de pessoas admitidas no Ambulatório Trans segundo características sociodemográficas. Distrito Federal, 2018-2022 (n=261)

Variáveis	Homens trans (n=149) n (%)	Mulheres trans (n=98) n (%)	Travestis (n=14) n (%)	Total (n=261) n (%)
**Faixa etária **(anos)				
18-29	120 (80,5)	44 (45,0)	8 (57,2)	172 (65,9)
30-39	21 (14,1)	26 (26,5)	3 (21,4)	50 (19,2)
40-49	8 (5,4)	21 (21,4)	2 (14,3)	31 (11,9)
>50	0 (0,0)	7 (7,1)	1 (7,1)	8 (3,0)
**Raça/cor da pele**				
Parda	68 (45,6)	40 (40,8)	7 (50)	115 (44,1)
Preta	23 (15,4)	20 (20,4)	2 (14,3)	45 (17,2)
Branca	49 (32,9)	34 (34,7)	4 (28,6)	87 (33,3)
Amarela	2 (1,3)	2 (2,0)	1 (7,1)	5 (1,9)
Indígena	2 (1,3)	1 (1,0)	0 (0,0)	3 (1,1)
Indisponível/ignorado	5 (3,4)	1 (1,0)	0 (0,0)	6 (2,5)
**Estado civil**				
Solteiro(a)	110 (73,8)	81 (82,7)	11 (78,6)	202 (77,4)
Casado(a)/união estável	28 (18,8)	10 (10,2)	2 (14,3)	40 (15,3)
Divorciado(a)	5 (3,4)	3 (3,1)	0 (0,0)	8 (3,1)
Viúvo(a)	0 (0,0)	1 (1,0)	1 (7,1)	2 (0,8)
Não quis responder	4 (2,7)	3 (3,1)	0 (0,0)	7 (2,7)
Indisponível/ignorado	2 (1,3)	0 (0,0)	0 (0,0)	2 (0,8)
**Orientação sexual**				
Heterossexual	108 (72,5)	71 (72,4)	4 (28,6)	183 (70,1)
Bissexual	21 (14,1)	12 (12,2)	4 (28,6)	37 (14,2)
Homossexual	5 (3,4)	6 (6,1)	3 (21,4)	14 (5,4)
Outro	15 (10,1)	9 (9,2)	3 (21,4)	27 (10,3)
Escolaridade				
Fundamental incompleto	4 (2,7)	12 (12,2)	0 (0,0)	16 (6,1)
Fundamental completo	5 (3,4)	4 (4,1)	0 (0,0)	9 (3,4)
Médio incompleto	15 (10,1)	27 (27,6)	3 (21,4)	45 (17,2)
Médio completo	46 (30,9)	23 (23,5)	3 (21,4)	72 (27,6)
Superior incompleto	51 (34,2)	17 (17,3)	5 (35,7)	73 (28,0)
Superior completo	22 (14,8)	10 (10,2)	2 (14,3)	34 (13,0)
Pós-graduação	4 (2,7)	3 (3,1)	1 (7,1)	8 (3,1)
Indisponível/ignorado	2 (1,3)	2 (2,0)	0 (0,0)	4 (1,5)
**Ocupação **				
Atividade remunerada	76 (51,0)	40 (40,8)	6 (42,9)	122 (46,7)
Comércio sexual	0 (0,0)	11 (11,2)	2 (14,3)	13 (5,0)
Estudante	50 (33,6)	20 (20,4)	2 (14,3)	72 (27,6)
Desempregado(a)	18 (12,1)	20 (20,4)	5 (35,7)	43 (16,5)
Outro	6 (4,0)	5 (5,1)	1 (7,1)	12 (4,6)
Indisponível/ignorado	7 (4,7)	10 (10,2)	0 (0,0)	17 (6,5)
**Benefícios assistenciais**				
Sim	11 (7,4)	9 (9,2)	4 (28,6)	24 (9,2)
Não	132 (88,6)	79 (80,6)	10 (71,4)	221 (84,7)
Indisponível/ignorado	6 (4,0)	10 (10,2)	0 (0,0)	16 (6,1)
**Situação de moradia**				
Imóvel alugado ou cedido	75 (50,4)	35 (35,7)	5 (35,7)	115 (44,1)
Imóvel próprio	43 (28,9)	24 (24,5)	3 (21,4)	70 (26,8)
Instituição de acolhimento/abrigo	1 (0,7)	5 (5,1)	0 (0,0)	6 (2,3)
Situação de rua	0 (0,0)	2 (2,0)	2 (14,3)	4 (1,5)
Indisponível/ignorado	30 (20,1)	32 (32,7)	4 (28,6)	66 (25,3)

Observou-se que 39,1% das pessoas conheceram o ambulatório por indicação de amigos (Tabela 2). As principais motivações para a busca de atendimento foram hormonização (76,6%) e acompanhamento multiprofissional (73,6%). Antes do atendimento, 56,7% já utilizavam hormônios – 37,8% sem receita médica (incluindo 6 das 10 travestis) e 35,2% com receita (especialmente homens trans). Modificações corporais já haviam sido realizadas por 48,0% (59,2% mulheres trans). A discriminação por gênero afetou 73,2% da amostra, e 25,3% haviam retificado o nome civil (nenhuma travesti).

**Tabela 2:** Distribuição de pessoas admitidas no Ambulatório Trans segundo características do percurso da transição de gênero. Distrito Federal, 2018-2022 (n=261)

Variáveis	Homens trans (n=149) n (%)	Mulheres trans (n=98) n (%)	Travestis (n=14) n (%)	Total (n=261)
**Conhecimento prévio do Ambulatório Trans**				
Por amigos ou conhecidos	69 (46,3)	27 (27,6)	6 (42,9)	102 (39,1)
Encaminhamento por serviço de saúde	25 (16,8)	24 (24,5)	1 (7,1)	50 (19,2)
Redes sociais	24 (16,1)	18 (18,4)	5 (35,7)	47 (18)
Centro de referência de assistência social ou centro de referência especializado de assistência social	15 (10,1)	15 (15,3)	1 (7,1)	31 (11,9)
Outros	10 (6,7)	12 (12,2)	1 (7,1)	23 (8,8)
Indisponível/ignorado	8 (5,4)	4 (4,1)	0 (0,0)	12 (4,6)
**Motivação para busca de atendimento**				
Hormonização	118 (79,2)	70 (71,4)	12 (85,7)	200 (76,6)
Acompanhamento multiprofissional	114 (76,5)	70 (71,4)	8 (57,1)	192 (73,6)
Acompanhamento médico	74 (49,7)	52 (53,1)	7 (50)	133 (51,0)
Acompanhamento endocrinológico	52 (34,9)	35 (35,7)	4 (28,6)	91 (34,9)
Cirurgias	47 (31,5)	38 (38,8)	5 (35,7)	90 (34,5)
Acompanhamento psiquiátrico	42 (28,2)	23 (23,5)	1 (7,1)	66 (25,3)
Outros	6 (4,0)	5 (5,1)	1 (7,1)	12 (4,6)
Indisponível/ignorado	0 (0,0)	1 (1,0)	0 (0)	1 (0,4)
**Modo de início da transição de gênero**				
Por conta própria	42 (28,2)	48 (49,0)	7 (50,0)	97 (37,2)
Ainda não iniciada	68 (45,6)	22 (22,4)	3 (21,4)	93 (35,6)
Acompanhamento profissional	29 (19,5)	17 (17,3)	2 (14,3)	48 (18,4)
Grupos da internet	7 (4,7)	5 (5,1)	0 (0,0)	12 (4,6)
Amigos	7 (4,7)	2 (2,0)	1 (7,1)	10 (3,8)
Indisponível/ignorado	5 (3,4)	8 (8,2)	1 (7,1)	14 (5,4)
Uso prévio de hormônios	71 (47,7)	67 (68,4)	10 (71,4)	148 (56,7)
**Forma de obtenção de hormônios**				
Farmácias sem receita médica	19 (26,8)	31 (46,3)	6 (60,0)	56 (37,8)
Farmácias com receita médica	25 (35,2)	16 (23,9)	1 (10,0)	42 (28,4)
Internet	9 (12,7)	2 (3,0)	0 (0,0)	11 (7,4)
Amigos/colegas de trabalho	5 (7,0)	0 (0,0)	0 (0,0)	5 (3,4)
Bombadeiras	0 (0,0)	3 (4,5)	0 (0,0)	3 (2,0)
Sistema Único de Saúde	0 (0,0)	1 (1,5)	0 (0,0)	1 (0,7)
Outro	1 (1,4)	2 (3)	0 (0,0)	3 (2,0)
Indisponível/ignorado	12 (16,9)	12 (17,9)	3 (30,0)	27 (18,2)
**Fonte de orientações**				
Profissional de saúde	23 (32,4)	17 (25,4)	1 (10,0)	41 (27,7)
Outras travestis/trans	2 (2,8)	12 (17,9)	2 (20,0)	16 (10,8)
Grupos da internet	6 (8,5)	5 (7,5)	0 (0,0)	11 (7,4)
Amigos	6 (8,5)	2 (3,0)	1 (10,0)	9 (6,1)
Outros	1 (1,4)	2 (3,0)	0 (0,0)	3 (2,0)
Indisponível/ignorado	33 (46,5)	29 (43,3)	6 (60,0)	68 (45,9)
**Modificação corporal prévia**	60 (40,3)	58 (59,2)	7 (50,0)	125 (47,9)
**Experiência de discriminação por gênero**	110 (73,8)	68 (69,4)	13 (92,9)	191 (73,2)
**Contexto da discriminação **				
Família	99 (66,4)	54 (55,1)	12 (85,7)	165 (63,2)
Espaços públicos	56 (37,6)	48 (49,0)	12 (85,7)	116 (44,4)
Emprego	23 (15,4)	23 (23,5)	3 (21,4)	49 (18,8)
Escola	18 (12,1)	14 (14,3)	2 (14,3)	34 (13,0)
Práticas religiosas	11 (7,4)	1 (1,0)	0 (0,0)	12 (4,6)
Serviços de saúde	2 (1,3)	3 (3,1)	0 (0,0)	5 (1,9)
Outros	2 (1,3)	6 (6,1)	0 (0,0)	8 (3,1)
**Apoio intradomiciliar**	90 (60,4)	33 (33,7)	3 (21,4)	126 (48,3)
**Apoio de outras redes **	66 (44,3)	26 (26,5)	7 (50,0)	99 (37,9)
**Retificação do nome**				
Sim	34 (22,8)	32 (32,7)	0 (0,0)	66 (25,3)
Não	114 (76,5)	66 (67,3)	14 (100,0)	194 (74,3)
Indisponível/ignorado	1 (0,7)	0 (0,0)	0 (0,0)	1 (0,4)

Houve predomínio no uso de serviços de urgência/emergência (56,7% no total) em comparação com UBS (21,0%); 69,0% não foram acessados(as) pela ESF; e 17,2% possuíam plano de saúde (Tabela 3). Verificaram-se altas prevalências de relato de doenças psiquiátricas (46,4%) e de uso de substâncias psicoativas (49,0%), com destaque para álcool, tabaco e maconha, as mais citadas. A infecção por HIV foi relatada por 9,8% entre mulheres trans e travestis, com nenhum caso em homens trans. Destacou-se a proporção de pessoas que declararam não se prevenir de HIV e outras IST na maioria das vezes, principalmente entre homens trans (37,6%). O uso de profilaxia pós-exposição e profilaxia pré-exposição foi baixo (5,1%), restrito a mulheres trans.

**Tabela 3:** Distribuição de pessoas admitidas no Ambulatório Trans segundo características de acesso a serviços de saúde e antecedentes clínicos. Distrito Federal, 2018-2022 (n=261)

Variáveis	Homens trans (n=149) n (%)	Mulheres trans (n=98) n (%)	Travestis (n=14) n (%)	Total (n=261)
**Serviço procurado ao adoecer**				
Emergência de hospital público	48 (32,2)	26 (26,5)	5 (35,7)	79 (30,3)
Unidade de pronto atendimento	43 (28,9)	25 (25,5)	1 (7,1)	69 (26,4)
Unidade básica de saúde	32 (21,5)	21 (21,4)	2 (14,3)	55 (21,1)
Emergência de hospital privado	20 (13,4)	17 (17,3)	5 (35,7)	42 (16,1)
Ambulatório ou consultório de clínica privada	21 (14,1)	17 (17,3)	3 (21,4)	41 (15,7)
Ambulatório de hospital público	10 (6,7)	15 (15,3)	0 (0,0)	25 (9,6)
Farmácia	11 (7,4)	6 (6,1)	1 (7,1)	18 (6,9)
Centro de especialidades ou policlínica pública	4 (2,7)	9 (9,2)	0 (0,0)	13 (5,0)
Profissional da equipe de saúde da família em casa	0 (0,0)	0 (0,0)	0 (0,0)	0 (0,0)
Outro	9 (6,0)	5 (5,1)	3 (21,4)	17 (6,5)
Indisponível/ignorado	14 (9,4)	16 (16,3)	1 (7,1)	31 (11,9)
**Acessado(a) pela estratégia saúde da família **				
Sim	15 (10,1)	9 (9,2)	0 (0,0)	24 (9,2)
Não	99 (66,4)	68 (69,4)	13 (92,9)	180 (69,0)
Não sabe	10 (6,7)	4 (4,1)	0 (0,0)	14 (5,3)
Indisponível/ignorado	25 (16,8)	17 (17,3)	1 (7,1)	43 (16,5)
**Última consulta médica **				
Nos últimos 12 meses	102 (68,5)	68 (69,4)	8 (57,2)	178 (68,2)
1-2 anos	11 (7,4)	10 (10,2)	2 (14,3)	23 (8,8)
2-3 anos	12 (8,0)	5 (5,1)	1 (7,1)	18 (6,9)
>3 anos	11 (7,4)	4 (4,1)	2 (14,3)	17 (6,5)
Nunca foi a médico ou serviço de saúde	1 (0,7)	1 (1,0)	0 (0,0)	2 (0,8)
Indisponível/ignorado	12 (8,0)	10 (10,2)	1 (7,1)	23 (8,8)
Beneficiário(a) de plano de saúde	22 (14,8)	20 (20,4)	3 (21,4)	45 (17,2)
**Doença preexistente**				
Doenças psiquiátricas	72 (48,3)	45 (45,9)	4 (28,6)	121 (46,4)
Hipertensão arterial	3 (2,0)	8 (8,2)	1 (7,1)	12 (4,6)
HIV	0 (0,0)	9 (9,2)	2 (14,3)	11 (4,2)
Obesidade	3 (2,0)	4 (4,1)	0 (0,0)	7 (2,7)
Sífilis	1 (0,7)	4 (4,1)	1 (7,1)	6 (2,3)
Asma	2 (1,3)	1 (1,0)	0 (0,0)	3 (1,1)
Diabetes	0 (0,0)	1 (1,0)	1 (7,1)	2 (0,8)
Dislipidemia	1 (0,7)	1 (1,0)	0 (0,0)	2 (0,8)
Neoplasias	0 (0,0)	1 (1,0)	0 (0,0)	1 (0,4)
Hepatite C	0 (0,0)	1 (1,0)	0 (0,0)	1 (0,4)
Outra infecção sexualmente transmissível (IST)	0 (0,0)	1 (1,0)	0 (0,0)	1 (0,4)
Hepatite B	0 (0,0)	0 (0,0)	0 (0,0)	0 (0,0)
Outras	69 (46,3)	34 (34,7)	4 (28,6)	107 (41,0)
Indisponível/ignorado	17 (11,4)	11 (11,2)	3 (21,4)	31 (11,9)
**Uso prévio de substâncias psicoativas**	73 (49,0)	47 (48,0)	8 (57,1)	128 (49,0)
**Testagem para IST nos últimos 12 meses**	48 (32,2)	54 (55,1)	9 (64,3)	111 (42,5)
**Forma de prevenção de IST**/HIV/aids				
Não se previne a maior parte das vezes	56 (37,6)	6 (6,1)	1 (7,1)	63 (24,1)
Preservativo	8 (5,4)	41 (41,8)	6 (42,9)	55 (21,1)
Profilaxia pré-exposição	0 (0,0)	5 (5,1)	0 (0,0)	5 (1,9)
Profilaxia pós-exposição	0 (0,0)	5 (5,1)	0 (0,0)	5 (1,9)
Outro método	15 (10,1)	13 (13,3)	2 (14,3)	30 (11,5)
Indisponível/ignorado	70 (47,0)	39 (39,8)	5 (35,7)	114 (43,7)

Não buscar atendimento na UBS associou-se significativamente à maior probabilidade de não ser acessado pela ESF (RP 1,36; IC95% 1,11; 1,66; p-valor<0,001) (Tabela 4). 

**Tabela 4:** Razões de prevalência (RP) brutas e intervalos de confiança de 95% (IC95%) de não acesso à estratégia saúde da família (ESF) entre pessoas admitidas no Ambulatório Trans, segundo características sociodemográficas, percurso de transição de gênero e condições de acesso a serviços de saúde. Distrito Federal, 2018-2022 (n=261)

Variáveis	Não acessado(a) pela ESF n (%)	RP (IC95%)	p-valor
Sociodemográficas			
Gênero			0,371^a^
Travestis	13 (100,0)	1,14 (1,08; 1,21)	
Outros	167 (87,4)	1,00	
**Faixa etária **(anos)			0,546^a^
18-39	152 (87,4)	0,94 (0,84; 1,05)	
>40	28 (93,3)	1,00	
**Raça/cor da pele**			0,052^a^
Pardos, brancos e outros	149 (90,3)	1,15 (0,97; 1,37)	
Pretos	29 (78,4)	1,00	
**Orientação sexual**			0,270^b^
Heterossexual	118 (86,1)	0,93 (0,85; 1,02)	
Outros	62 (92,5)	1,00	
Escolaridade			0,053^a^
Médio incompleto	38 (97,4)	1,13 (1,05; 1,23)	
Outros	140 (85,9)	1,00	
**Percurso de transição de gênero**			
**Modo de início da transição de gênero**			0,270^b^
Por conta própria	78 (91,8)	1,07 (0,97; 1,18)	
Outros	102 (85,7)	1,00	
**Uso prévio de hormônios**			0,630^b^
Não	71 (86,6)	0,96 (0,87; 1,07)	
Sim	106 (89,8)	1,00	
**Forma de obtenção de hormônio**			0,124^a^
Sem receita médica	54 (94,7)	1,13 (0,96; 1,33)	
Com receita médica	26 (83,9)	1,00	
**Apoio de outras redes**			0,449^a^
Não	60 (90,9)	1,06 (0,95; 1,19)	
Sim	71 (85,5)	1,00	
**Condições de acesso a serviços de saúde**			
**Última consulta médica **			0,109^b^
Últimos 12 meses	122 (85,3)	0,90 (0,82; 0,99)	
Outros	55 (94,8)	1,00	
**Beneficiário(a) de plano de saúde**			0,052^a^
Sim	38 (97,4)	1,13 (1,04; 1,23)	
Não	131 (86,2)	1,00	
**Busca por atendimento na unidade básica de saúde quando adoece**			<0,001^b^
Não	149 (93,7)	1,36 (1,11; 1,66)	
Sim	31 (68,9)	1,00	
**Hipertensão arterial preexistente**			0,611^a^
Sim	10 (100,0)	1,14 (1,08; 1,2)	
Não	170 (87,6)	1,00	

^a^Teste exato de Fisher; ^b^Teste do qui-quadrado de Pearson.

O modelo logístico ajustado demonstrou boa capacidade discriminativa (ROC 0,847) e adequação metodológica, confirmada pelo teste de Hosmer-Lemeshow (p-valor 0,963); ausência de multicolinearidade (teste de fator de inflação da variância 1); análise de valores atípicos; e distribuição adequada dos resíduos. A busca por atendimento na UBS reduziu em 86,0% a chance de não vinculação à ESF (OR 0,14; IC95% 0,05; 0,37; p-valor<0,001) (Tabela 5). O intercepto elevado (OR 25,27; IC95% 2,39; 266,77; p-valor 0,015) indicou alta probabilidade basal de não ter sido acessado(a) pela ESF. Outras variáveis não mostraram significância estatística nas Tabelas 4 e 5. 

**Tabela 5:** Razões de chances (*odds ratio*, OR) ajustadas e intervalos de confiança de 95% (IC95%) de não acesso à estratégia saúde da família entre pessoas do Ambulatório Trans, segundo características sociodemográficas, percurso de transição de gênero e condições de acesso a serviços de saúde. Distrito Federal, 2018-2022 (n=261)

Variáveis	Coeficiente estimado (β)	Erro-padrão	p-valor	OR (IC95%)
Intercepto	3,23	1,20	0,015	25,27 (2,39; 266,77)
**Pardos e outros (versus pretos)**	0,94	0,55	0,094	2,55 (0,87; 7,45)
**Nível de escolaridade diferente de médio incompleto**	-1,86	1,07	0,086	0,16 (0,02; 1,26)
**Início da transição de gênero por conta própria (vs. outras formas)**	0,68	0,52	0,194	1,98 (0,71; 5,50)
**Última consulta médica >12 meses (vs. <12 meses)**	0,82	0,68	0,235	2,28 (0,60; 8,63)
**Busca por atendimento na unidade básica de saúde quando adoece **(vs. não)	-1,95	0,49	<0,001	0,14 (0,05; 0,37)

## Discussão

Os resultados revelaram que mais da metade das pessoas admitidas no Ambulatório Trans do Distrito Federal eram homens trans, seguidos por mulheres trans e travestis, com predominância de pessoas jovens, negras, solteiras, heterossexuais e com até o ensino médio completo. Os principais desafios identificados abrangeram baixa proporção de retificação documental, diferentes contextos de discriminação, início da transição de gênero sem suporte profissional, alta prevalência de doenças psiquiátricas e concentração de atendimentos no serviço de emergência em detrimento da APS. A frequência prévia em UBS aumentou a vinculação com a ESF, o que reduziu a probabilidade de desassistência. 

Evidenciaram-se ainda disparidades entre as categorias de gênero estudadas, sobretudo entre as travestis. Apesar de constituírem o menor percentual do total, estas apresentaram as condições socioeconômicas mais precárias. Essa realidade foi confirmada por um estudo do Rio de Janeiro que, apesar das diferenças metodológicas, também identificou as travestis como o grupo com os piores indicadores de renda, escolaridade e acesso à saúde [13].

No Brasil, a ausência de dados oficiais por identidade de gênero dificulta a caracterização precisa dessa população. Em 2021, as estimativas disponíveis sugerem que 1% da população adulta se identifica como pessoas trans binárias, com distribuição equilibrada entre homens e mulheres [14]. Contudo, tais estimativas não distinguem entre as categorias de mulheres trans e travestis.

Em comparação com dados nacionais, identificou-se uma sobrerrepresentação de jovens nesta amostra (mais de duas vezes maior que a da população geral brasileira), enquanto a proporção de pessoas negras aproximou-se da composição nacional, o que representou mais da metade do total [15]. A possível interseccionalidade dessas variáveis intensifica as disparidades em saúde, como no caso de pessoas jovens trans negras no Canadá, que, em 2023, apresentaram o dobro de probabilidade de não receber cuidados físicos necessários em comparação com seus pares brancos [16]. 

A proporção de pessoas trans com ensino superior completo deste estudo foi 23% menor que a da população geral de 25 anos ou mais [15]. A exclusão escolar e as barreiras estruturais na trajetória acadêmica de pessoas trans acarretam evasão educacional, limitam oportunidades profissionais e perpetuam a marginalização socioeconômica na fase adulta [17].

A amostra investigada apresentou menor percentual de heterossexuais, quando comparada com a população geral [18]. Identidade de gênero e orientação sexual são dimensões distintas, não fixas, e podem apresentar variações ao longo da vida [19]. Tanto pessoas trans quanto cisgênero (cis) podem ter qualquer orientação sexual, o que mostra a pluralidade das experiências identitárias humanas. 

A alta frequência de discriminação por identidade de gênero foi confirmada por pesquisa realizada em cinco capitais brasileiras com mulheres trans e travestis [20]. O aumento dessa forma de discriminação foi associado ao histórico de violência e às características sociodemográficas de maior vulnerabilidade. 

A maior demanda por hormonização e acompanhamento multiprofissional em comparação com as cirurgias pode ser compreendida pelo princípio da autodeterminação de gênero, que decorre de processos identitários construídos a partir das experiências de vida. Isso inclui a hormonização como recurso essencial de afirmação de gênero, independentemente da necessidade de procedimentos cirúrgicos [21].

A prática de automedicação hormonal nessa população já havia sido registrada em pesquisa brasileira [8]. O predomínio do início da transição de gênero sem acompanhamento profissional (especialmente entre mulheres trans e travestis) contrasta com achados de estudo chileno [22], no qual 58% das pessoas trans em uso de hormônios afirmaram obtê-los por meio de profissionais de saúde. A desigualdade regional brasileira na oferta de serviços especializados [10] dificulta o acesso dessa população a cuidados adequados. No Brasil, o fato de a testosterona ser um medicamento controlado pode restringir a hormonização sem atendimento profissional entre homens trans, uma vez que esse procedimento constitui uma das principais demandas de modificação corporal desse grupo [23].

A falta de retificação do nome civil impacta negativamente os indicadores socioeconômicos e leva as pessoas a postergarem a busca por serviços de saúde [24]. Essa violência estrutural e institucional, ao se interseccionar com outras formas de opressão, constitui uma barreira a diferentes direitos sociais. Na Argentina [25], mulheres trans discriminadas por profissionais de saúde tiveram três vezes mais chances de evitar serviços de saúde que as não discriminadas. 

A baixa cobertura de planos de saúde – inferior às médias do Distrito Federal (33%) e nacional (25%) [26] – indicou que a maior parte das pessoas investigadas é usuária do SUS. Apesar do aumento da cobertura da ESF no Distrito Federal de 34% em 2017 para 77% em 2023 [27], tal expansão mostrou-se insuficiente para superar lacunas assistenciais verificadas neste estudo, no âmbito da APS. 

No que tange a doenças preexistentes, observaram-se algumas semelhanças com pesquisa norte-americana, na qual mulheres trans relataram piores resultados de saúde em comparação com homens trans, com exceção da depressão [28]. Embora a presente pesquisa não tenha especificado os tipos de doenças psiquiátricas, a prevalência de autorrelato dessas condições foi quatro vezes superior à prevalência de triagem positiva para depressão na população adulta brasileira [29]. Para além dos cuidados de afirmação de gênero, é necessária uma abordagem que incorpore integralmente a saúde mental, com enfrentamento à discriminação no contexto individual e social [30].

O uso de substâncias psicoativas por mulheres trans e travestis apresentou percentual semelhante ao constatado em pesquisa brasileira anterior [31], que apontou álcool, tabaco e maconha como as substâncias mais consumidas. O uso múltiplo de substâncias, ao associar-se a maior violência, desemprego e trabalho informal, configura-se como possível estratégia de enfrentamento ao sofrimento e à marginalização.

A baixa adesão à profilaxia pré-exposição entre mulheres trans é corroborada por meio de achados de estudo nacional [32], que mostra apenas 6% de pessoas trans em uso dessa profilaxia no Brasil (vs. 94% de pessoas cis). A prevenção do HIV e de outras IST demanda abordagens integradas, que combinem estratégias diversificadas e adaptações específicas para cada grupo de gênero estudado. 

Esta pesquisa apresentou limitações, principalmente devido ao seu desenho transversal, que impede a inferência de relações de causalidade ou temporais. Além disso, o autorrelato pode comprometer a confiabilidade de variáveis sensíveis e introduzir vieses de informação. 

A amostra, de caráter não probabilístico, incluiu exclusivamente pessoas trans e travestis atendidas no ambulatório, o que configura um viés de seleção por não incorporar aquelas que ainda não acessaram o serviço. Pessoas não binárias e de outras identidades de gênero, embora atendidas pelo serviço, também não foram contempladas no estudo. Contudo, buscou-se caracterizar detalhadamente os demais grupos que tiveram acesso ao serviço.

O contexto do Distrito Federal, com sua estrutura particular de serviços, também pode diferir de outras realidades e limitar a generalização dos resultados. Apesar de o ambulatório registrar dados não captados por outros serviços, a limitação desta pesquisa às variáveis de registro rotineiro pode não abranger aspectos sociais e culturais importantes. Altas proporções de dados indisponíveis ou ignorados de algumas variáveis, como renda individual e familiar, precisaram ser excluídas por restringirem a análise. A dimensão amostral modesta pode ter sido insuficiente para detectar diferenças significativas entre os grupos, embora tenha permitido análises descritivas e de associação.

Verificou-se ainda expressiva sub-representação de travestis e sobrerrepresentação de homens trans, o que limita análises específicas para esses grupos. A apresentação estratificada dos dados das travestis foi mantida como estratégia metodológica e política para visibilizar sua especificidade identitária e confrontar sua histórica invisibilidade social, ainda que o termo “trans” seja frequentemente utilizado na literatura em seu sentido amplo para englobar diversas identidades de gênero. A investigação também não analisou a resolubilidade de demandas mais amplas das pessoas atendidas nem investigou detalhadamente a vinculação com serviços da rede de cuidado e outras necessidades em saúde. 

Apesar das limitações, este estudo representou uma contribuição relevante, ao servir como base exploratória para pesquisas futuras, inclusive qualitativas. 

Em conclusão, os resultados mostraram iniquidades sociodemográficas, desigualdades na trajetória de transição de gênero e no acesso a serviços de saúde, com subutilização da APS. O vínculo com a UBS mostrou-se protetor, o que reforça a ESF como modelo para o cuidado integral. Além de articular a APS com os serviços especializados, é preciso expandir, de forma equitativa, a cobertura da ESF no Distrito Federal para reduzir a demanda por serviços de urgência e implementar medidas preventivas para doenças psiquiátricas e outras morbidades.

Os achados também revelaram a necessidade de políticas intersetoriais em âmbito distrital e nacional para assegurar direitos sociais plenos. Essas ações, somadas a campanhas antidiscriminatórias, são essenciais para mitigar barreiras e garantir equidade no acesso, em consonância com os princípios do SUS. 

## Data Availability

Para proteger a privacidade das pessoas participantes e garantir o sigilo, somente os dados consolidados estão disponíveis. O banco de dados completo permanece restrito devido ao caráter sensível e individualizado das informações, que, mesmo após anonimização, podem apresentar riscos de identificação.
